# The Floral Repressor *GmFLC-like* Is Involved in Regulating Flowering Time Mediated by Low Temperature in Soybean

**DOI:** 10.3390/ijms21041322

**Published:** 2020-02-15

**Authors:** Jing Lyu, Zhandong Cai, Yonghong Li, Haicui Suo, Rong Yi, Shuai Zhang, Hai Nian

**Affiliations:** 1 State Key Laboratory for Conservation and Utilization of Subtropical Agro-Bioresources, South China Agricultural University, Guangzhou 510642, China; bylvjing@126.com (J.L.); zdcai@stu.scau.edu.cn (Z.C.); yirongscau@163.com (R.Y.); 2 School of Applied Chemistry and Biological Technology, Postdoctoral Innovation Practice Base, Shenzhen Polytechnic, Shenzhen 518055, China; liyongh@szpt.edu.cn; 3 Crop Research Institute, Guangdong Academy of Agriculture, Guangzhou 510642, China; suohaicui@163.com; 4 Key Laboratory of Plant Molecular Breeding, South China Agricultural University, Guangzhou 510642, China

**Keywords:** soybean, flowering time, low temperature, *GmFLC-like*, *GmFT*

## Abstract

Soybean is an important crop that is grown worldwide. Flowering time is a critical agricultural trait determining successful reproduction and yields. For plants, light and temperature are important environmental factors that regulate flowering time. Soybean is a typical short-day (SD) plant, and many studies have elucidated the fine-scale mechanisms of how soybean responds to photoperiod. Low temperature can delay the flowering time of soybean, but little is known about the detailed mechanism of how temperature affects soybean flowering. In this study, we isolated *GmFLC-like* from soybean, which belongs to the *FLOWERING LOCUS C* clade of the MADS-box family and is intensely expressed in soybean leaves. Heterologous expression of *GmFLC-like* results in a delayed-flowering phenotype in Arabidopsis. Additional experiments revealed that *GmFLC-like* is involved in long-term low temperature-triggered late flowering by inhibiting *FT* gene expression. In addition, yeast one-hybrid, dual-luciferase reporter assay, and electrophoretic mobility shift assay revealed that the GmFLC-like protein could directly repress the expression of *FT2a* by physically interacting with its promoter region. Taken together, our results revealed that GmFLC-like functions as a floral repressor involved in flowering time during treatments with various low temperature durations. As the only the *FLC* gene in soybean, *GmFLC-like* was meaningfully retained in the soybean genome over the course of evolution, and this gene may play an important role in delaying flowering time and providing protective mechanisms against sporadic and extremely low temperatures.

## 1. Introduction

Understanding the molecular mechanism driving the change from vegetative to reproductive growth is crucial for maximizing the yield of seed crops in a given environment. As one of the most important traits, flowering is considered the developmental transition from juvenile to adult phase, and the flowering process is also regulated by various internal signals and environmental cues. Five major pathways controlling flowering time, which include the photoperiod, vernalization, autonomous, ageing, and gibberellin pathways, have been identified in Arabidopsis (*Arabidopsis thaliana*) on the basis of previous studies [1,2], and the photoperiod and vernalization pathways are triggered by environmental cues such as light and temperature [3,4].

Light, including the duration, quality, and intensity, is one of the most important environmental cues modulating flowering at multiple levels [5]. According to the day length, plants can be ranked into three categories: long-day (LD) plants, that proceed to flowering when the day length exceeds a threshold value; short-day (SD) plants, that flower in response to a shortening photoperiod; and day-neutral plants, whose flowering does not rely on the day length [6]. The change in light quality triggers multiple responses referred to as the “shade avoidance syndrome”. Plants focus on stem elongation instead of leaf expansion during the shade avoidance period and finally achieve the objective of early flowering [7]. Light intensity, one of most important parameters for light, has independent functions in the regulation of flowering time by accelerating the transition from juvenile to adult phase in Arabidopsis and other higher plants [5].

Apart from light, the temperature acts as another environmental signal affecting flowering time in two distinguishing ways [8]. The first way is that some species must undergo a prolonged cold period to obtain the capability of floral transition, and this cold-accumulation is known as vernalization [9,10]. Second, some species respond to the ambient temperature pathway, referring to non-stressful and physiological temperatures perceived by plants at the vegetative stage [8,11]. For some winter annual, biennial, and perennial plants, the vernalization requirements (at 1 to 7 °C) are absolutely necessary and determine reproductive success in spring or early summer. Previously, extensive genetic and molecular approaches exploring the underlying mechanism of vernalization in the model species, *Arabidopsis thaliana*, have identified that *FLOWERING LOCUS C* (*FLC*) is a major gene regulating vernalization requirements and responses. Briefly, vernalization represses the expression of *FLC*, a floral repressor [12,13]. Previous studies have shown that the vernalization pathway genes *FRIGIDA* (*FRI*) and *VERNALIZATION INSENSITIVE 3* (*VIN3*) are key genes responsible for regulating *FLC* expression [14,15]. While the loss-of-function mutations in *FRI* displayed an early-flowering phenotype in Arabidopsis, this evolving phenotype has no need for the vernalization pathway [16,17]. *VIN3*, which is transiently induced by low temperature, is essential for *FLC* repression [18,19]. In addition, the epigenetic regulation is also involved in silencing *FLC*, including chromatin modification, *COLD INDUCED LONG ANTISENSE INTRAGENIC RNA* (*COOLAIR*), and *COLD ASSISTED INTRONIC NONCOLDING RNA* (*COLDAIR*) [20,21,22,23,24]. In Arabidopsis, the flowering genes *FLOWERING LOCUS T* (*FT*) and *SOC1* are positive regulators of flowering, and the late-flowering phenotype could be due to a decrease in the expression level of *FT* and *SUPPRESSOR OF OVEREXPRESSION OF CONSTANTS 1* (*SOC1*) by the FLC target [25,26,27,28].

The function of *FLC* in the response to vernalization was conserved in some species, such as *Brassica napus*, *Brassica rapa*, *Sinapis alba*, *Capsella rubella*, *Beta vulgaris*, and *Cichorium intybus*. These *FLC-like* genes, whose function is consistent with that of *AtFLC*, act as floral repressors and are downregulated in response to cold [29,30,31,32,33,34]. However, *VERNALIZATION 1* and *2* (*VRN1* and *VRN2*), the homologues of *APETALA 1* (*AP1*) and *FT* in Arabidopsis, respectively, are responsible for the vernalization requirements instead of *FLC* in wheat and barley [35]. These findings indicate that different genes account for vernalization requirements in different species.

In contrast to the detailed understanding of vernalization, research in connection with the fine mechanism by which ambient temperatures affect flowering time has just started. In Arabidopsis, elevating the growth temperature from 23 to 27 °C dramatically facilitates flowering under SD conditions; this phenotype is a primary attribute of the change in *FT* expression levels [36]. In contrast, for some species such as *Boechera stricta* and *Chrysanthemum morifolium*, when growth temperatures moderately increase, the plants exhibit delayed flowering [37,38].

Soybean (*Glycine max*) is one of the most important crops worldwide for its nutritional qualities and oil content, and its flowering time is a critical agricultural trait determining successful reproduction and yields. Hence, identifying the functions of key soybean genes is of great importance for the genetic improvement of crops. Soybean is a typically SD plant whose flowering and maturation are strictly controlled by the photoperiod [39], and many studies have explored the fine-scale mechanisms of how soybean responds to the photoperiod [40,41,42,43]. In addition, although soybean is a non-vernalized plant, the temperature is also a major environmental factor affecting its flowering time. Low temperature could delay the flowering time of soybean, but little is known about the detailed mechanism of how temperature affects soybean flowering until now. In our previous study, we found that overexpression of *AtDREB1A* in soybean caused clearly delayed flowering [44]. qRT-PCR analyses of the expression of flowering time genes related to the vernalization pathway showed that *Glyma11g13220* (*GmVRN1-like*) and *Glyma05g28130* (designated as *GmFLC-like*) were strongly upregulated in the *DEHYDRATION RESPONSE ELEMENT B1A* (*AtDREB1A*)-overexpressing soybean [45]. Hence, we speculate that these genes may mainly account for the phenotype. Fortunately, we confirmed that the vernalization pathway gene *Glyma11g13220* plays crucial roles in regulating flowering time [46]. In this study, we isolated *Glyma05g28130* from soybean, which is intensely expressed in soybean leaves and is involved in long-term low temperature-triggered late flowering, belonging to the FLC clade of the MADS-box family. Additional experiments revealed that heterologous expression of *GmFLC-like* results in the phenotype of delayed flowering by inhibiting *FT* genes’ transcription in Arabidopsis. In addition, yeast one-hybrid (Y1H), dual-luciferase reporter assay, and electrophoretic mobility shift assay (EMSA) revealed that GmFLC-like protein could directly repress the expression of *FT2a* by physically interacting with its promoter region. In brief, our findings underline the importance of *GmFLC-like* in the soybean response to low temperature and highlight the role of this gene as a floral repressor in delaying flowering time and providing protective mechanisms against sporadic and extreme low temperatures.

## 2. Results

### 2.1. Glyma05g28130 Is a Homologue of AtFLC

**Figure 1:**
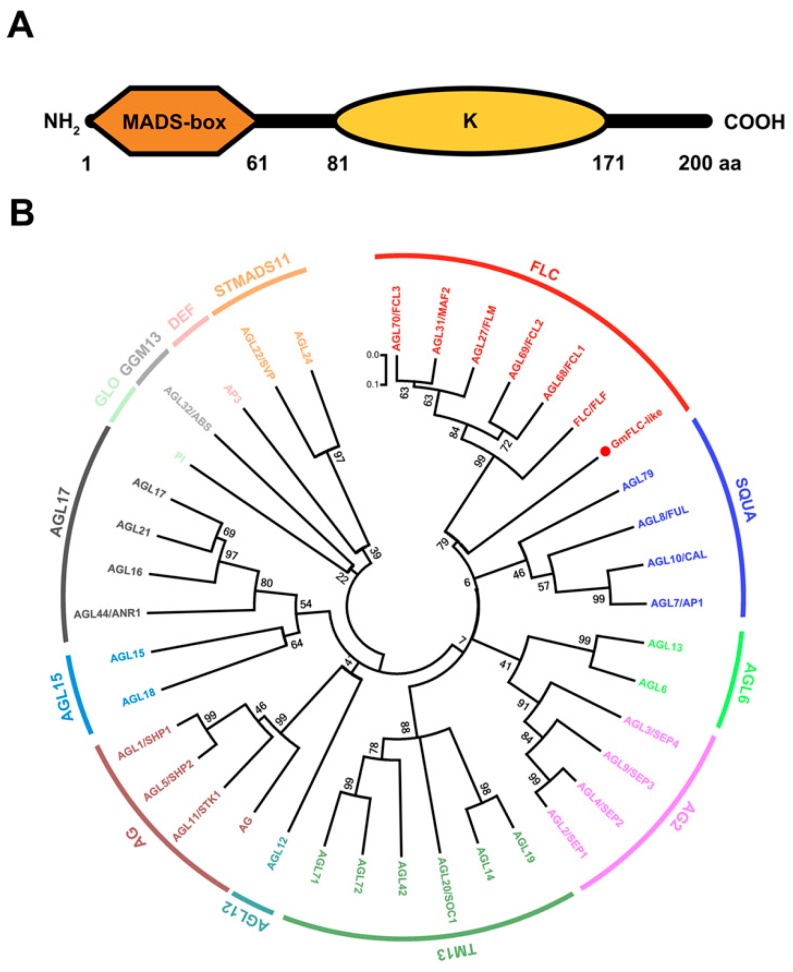
Sequence analyses of *GmFLC-like*. (**A**) Structure of GmFLC-like conserved domains, MADS-box (1–61 aa) and K (keratin-like) domains (81–171 aa). (**B**) Phylogenetic analysis of the GmFLC-like and MIKC-type MADS-box proteins from Arabidopsis. The phylogenetic tree was constructed using the neighbor-joining method with 1000 bootstrap replicates by MEGA 7.0 software, and bootstrap values are shown at the nodes. The locus of Arabidopsis MADS-box proteins as follows: AG (AT4G18960), AGL1/SHP1 (AT3G58780), AGL2/SEP1 (AT5G15800), AGL3/SEP4 (AT2G03710), AGL4/SEP2 (AT2G21970), AGL5/SHP2 (AT2G42830), AGL6 (AT2G45650), AGL7/AP1 (AT1G69120), AGL8/FUL (AT5G60910), AGL9/SEP3 (AT1G24260), AGL10/CAL (AT1G26310), AGL11/STK1 (AT4G09960), AGL12 (AT1G71692), AGL13 (AT3G61120), AGL14 (AT4G11880), AGL15 (AT5G13790), AGL16 (AT3G57230), AGL17 (AT2G22630), AGL18 (AT3G57390), AGL19 (AT4G22950), AGL20/SOC1 (AT2G45660), AGL21 (AT4G37940), AGL22/SVP (AT2G22540), AGL24 (AT4G24540), AGL27/FLM (AT1G77080), AGL31/MAF2 (AT5G65050), AGL32/ABS (AT5G23260), AGL42 (AT5G62165), AGL44/ANR1 (AT2G14210), AGL68/FCL1 (AT5G65080), AGL69/FCL2 (AT5G65070), AGL70/FCL3 (AT5G65060), AGL71 (AT5G51870), AGL72 (AT5G51860), AGL79 (AT3G30260), PI (AT5G20240), P3 (AT3G54340), FLC/FLF (AT5G10140).

**Figure 2:**
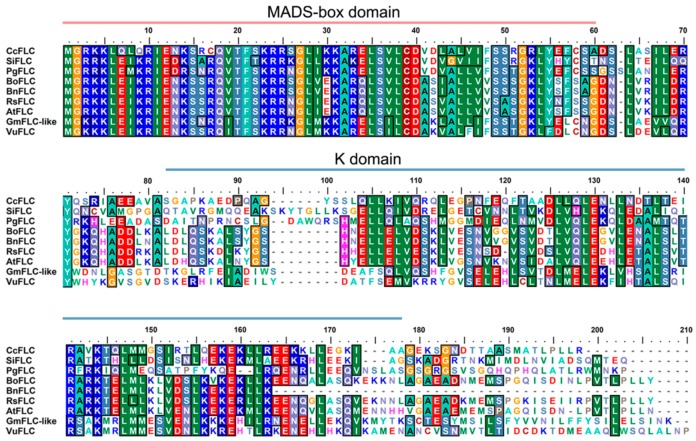
Sequence alignment of the GmFLC-like protein and other FLC proteins from other species. The pink line marks the MADS-box domain (1–61 aa), and the light blue line labels the K domain (81–171 aa). GenBank accession numbers are as follows: *Glycine max* GmFLC-like (MK913903), *Arabidopsis thaliana* AtFLC (NP_196576), *Vigna unguiculata* VuFLC (XP_027918635), *Punica granatum* PgFLC (OWM76224v), *Citrus clementina* CcFLC (XP_024041198), *Brassica oleracea* BoFLC (AHH30724), *Sesamum indicum* SiFLC (XP_011086821), *Raphanus sativus* RsFLC (AJN00653), *Brassica napus* BnFLC (AFU61576).

Based on previous results from our laboratory, Glyma05g28130 plays crucial roles in modulating flowering time in soybean [45]. To identify the functions of Glyma05g28130 in regulating flowering time, we cloned the gene from the soybean cultivar “Huachun 5” referring to the sequence found in the Phytozome database [47]. The results showed that the cDNA sequence of Glyma05g28130 is 1513 bp in length, contains a 603 bp ORF and encodes 200 amino acid residues. Glyma05g28130 has a predicted DNA-binding MADS-domain in the N-terminus, followed by the K (keratin-like) regions, and the domains were predicted at amino acid residues 1–61 and 71–181, respectively (Figure 1A). In Arabidopsis, the ancient MIKC-type MADS-box genes were further classified into 13 distinctive subfamilies based on their phylogeny, namely, AGL2, AGL6, SQUA, AGL12, FLC, TM3, AGL17, AG, AGL15, DEF, GLO, GGM13, and STMADS11 genes [48,49,50]. Phylogenetic analyses revealed that Glyma05g28130 fell within the FLC clade of the Arabidopsis MIKC-type MADS-box family and shared a close relationship with AtFLC (AT5G10140) (Figure 1B). Hence, the gene corresponding to Glyma05g28130 was named GmFLC-like. In addition, we compared the amino acid sequence of GmFLC-like with FLC homologues of other species. The MADS-box domain sequence of GmFLC-like shared high conservation among different species, whereas conservation of the K domain was much weaker (Figure 2).

### 2.2. Expression Profile and Biochemical Properties of GmFLC-like

**Figure 3:**
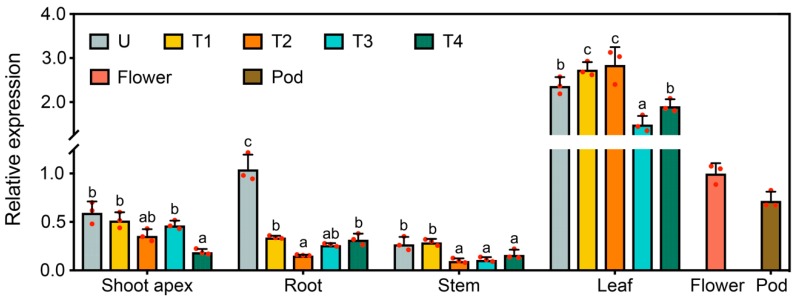
Expression analysis of *GmFLC-like* in different organs of soybean during multiple developmental stages under short-day (SD) conditions. U, the unifoliate period; T1, the first trifoliate period; T2, the second trifoliate period; T3, the third trifoliate period; T4, the fourth trifoliate period; Shoot apex (including apical meristem and immature leaves); Pod (14 days after flowering). *Gmβ-tubulin* (*Glyma20 g27280*) was used as an internal control. Error bar represents the means of three biological replicates, and the letters indicate significant differences according to Duncan’s multiple range test (*p* < 0.05).

In an attempt to understand whether *GmFLC-like* expression has tissue specificity, we analyzed *GmFLC-like* transcripts in multiple tissues, including shoot apexes, roots, stems, fully expanded leaves, flowers, and pods, during the soybean development process under SD conditions by qRT-PCR. The expression of *GmFLC-like* was greater in the leaves than in the flowers and pods and was lowest in the stems. In the shoot apex and root, the transcript of *GmFLC-like* was higher in the unifoliate period than in the other developmental stages (Figure 3).

**Figure 4:**
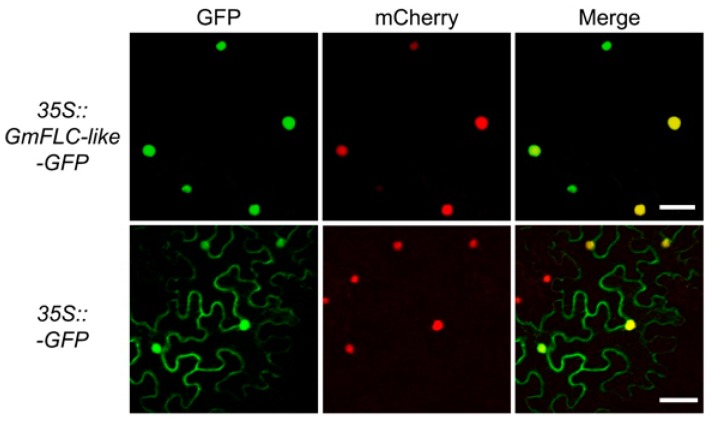
Subcellular localization of GmFLC-like protein in tobacco leaves. GFP fused to the C-terminal region of GmFLC-like, and the fusion protein was driven by 35S promoter. A mCherry labeled fusion protein (NF-YA4-mCherry) was used as a nuclear marker driven by 35S, and *35S::GFP* was used as negative control. At 3 days after infiltration, the fluorescence signals (GFP and mCherry) were visualized by confocal microscopy, and the excitation wavelengths for GFP and mCherry were 488 and 543 nm, respectively. Scale bar, 50 μM.

To determine the subcellular localization of GmFLC-like, we expressed a GmFLC-like-GFP protein together with a mCherry-labelled nuclear marker protein (NF-YA4-mCherry) in *N. benthamiana* leaves, and both proteins were driven by the 35S promoter. We observed that the GFP fluorescence signal pattern was consistent with the localization of the nuclear marker protein (mCherry) in the nucleus, indicating that GmFLC-like is a nuclear-localized protein (Figure 4). Similarly, transient expression of *GmFLC-like* in Arabidopsis protoplasts also confirmed that GmFLC-like is a nuclear protein (Figure S1).

### 2.3. Overexpression of GmFLC-like Caused Late Flowering in Arabidopsis

**Figure 5:**
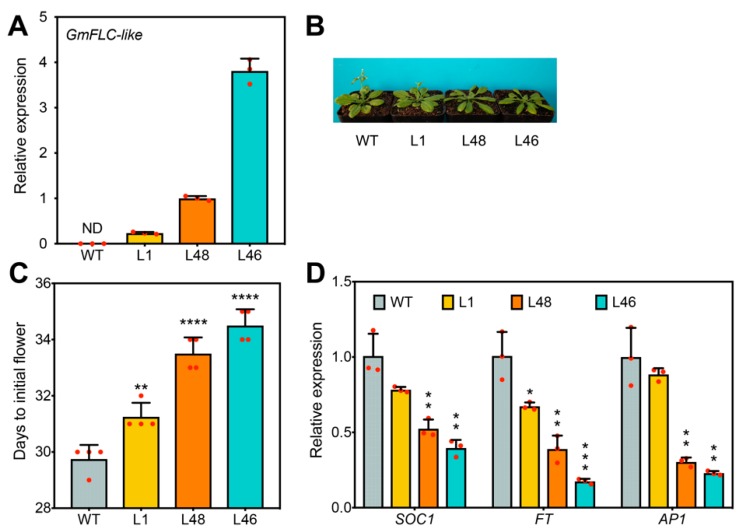
Ectopic expression of *GmFLC-like* in Arabidopsis. (**A**) Expression detection of *GmFLC-like* in transgenic plants. ND, not detected. (**B**) Phenotypic display of transgenic (L1, L46, and L48) and WT (Col-0) plants. The flowering phenotype of the *GmFLC*-like gene overexpression lines and WT plants were photographed at 5 weeks after sowing. Plants were grown at a growth chamber at 22 ± 1 °C under long-day (LD) conditions (16 h of light/8 h of dark). (**C**) Statistical analysis of days until the initial flowering of transgenic and WT (Col-0) plants. (**D**) Expression levels of flowering time genes in Arabidopsis. Arabidopsis *β-tubulin* (*AT5G62690*) was used as a negative control. Error bar represents the means of three biological replicates. Significant differences according to the *t*-test are denoted as follows: * *p* < 0.05, ** *p* < 0.01, *** *p* < 0.001, **** *p* < 0.0001. WT means the wild-type of Arabidopsis; L1, L46, and L48 refer to independent transgenic lines.

To investigate the biological function of *GmFLC-like* in regulating flowering time, we overexpressed *GmFLC-like* in Arabidopsis (Col-0), and the transcript abundance of *GmFLC-like* in the transgenic lines was confirmed using qRT-PCR (Figure 5A). Compared with the WT plants, the plants overexpressing *GmFLC-like* (L46 and L48) exhibited a clear delayed-flowering phenotype (Figure 5B). At 30 days, flower buds emerged in WT plants, while flower bud emergence of L46 and L48 plants was observed at days 34 and 35, respectively (Figure 5C). In addition, we also evaluated the expression of key downstream genes that are linked to flowering time by qRT-PCR analysis. The results indicated that, compared with the levels in the WT, transcript levels of floral activators, *FT*, *SOC1*, and *AP1*, in transgenic Arabidopsis (L46 and L48) decreased significantly (Figure 5D). Additional evidence indicated that the transgenic lines (L1, L48, and L46) displayed lower germination rates than the WT plants (Figure S2). Overall, *GmFLC-like* offers a similar function to *AtFLC*, and both of them function as a floral repressor.

### 2.4. *GmFLC-like* Is Responsive to the Photoperiod and Low Temperature

To further understand *GmFLC-like* potential functions, we analyzed the putative *cis*-acting elements in its promoter region (1500 bp sequence upstream of the start codon) by using the PlantCARE database. Different *cis*-acting regulatory elements involved in the light response, hormones, and development as well as abiotic stress were found (Table 1). Light response elements included AE-box, CATT-motif, G-box, TCT-motif, AT1-motif, Box-4, and so on. Hormone- and development-related elements included ABRE, GARE, TCA, and so on. HSE, MBS, ARE, and CE3, associated with abiotic stress responses, were also identified in the *GmFLC-like* upstream region. A variety of *cis*-acting regulatory elements in the *GmFLC-like* upstream region implies that the gene may be regulated by endogenous and external environmental signals.

**Figure 6:**
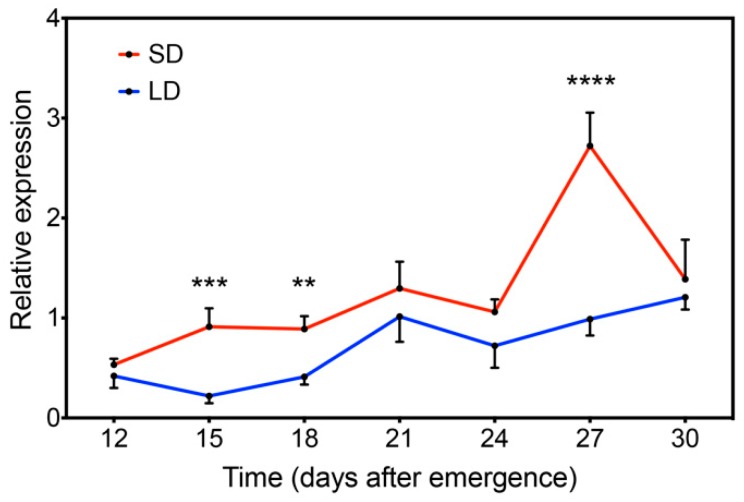
Expression analysis of *GmFLC-like* under SD and LD conditions at 12, 15, 18, 21, 24, 27, and 30 DAE (days after emergence). All seedlings were grown under SD conditions for 10 DAE, and then a portion of the seedlings were transferred to LD conditions. Fully expanded trifoliate leaves were sampled at the appointed time from three individual plants growing under SD and LD conditions. Significant differences according to the *t*-test are denoted as follows: ** *p* < 0.01, *** *p* < 0.001, **** *p* < 0.0001.

Based on many light-responsive *cis*-elements found in the *GmFLC-like* promoter region, we focused on the *GmFLC-like* function in response to the photoperiod (Table 1). Here, we chose the soybean variety “Huachun 5” as the research material, which is sensitive to the photoperiod because its flower buds emerge earlier under SD conditions [46]. Surprisingly, *GmFLC-like* functions as a floral inhibitor, but it displays a high transcript level under SD conditions. Further investigation concluded that *GmFLC-like* expression was markedly high under SD conditions at 15, 18 DAE (days after emergence), and showed the highest peak at 27 DAE, while *GmFLC-like* expression did not change outstandingly during the same period under the LD treatment (Figure 6). We suspect that the photoperiod pathway plays a leading role in the flowering regulation of soybean variety “Huachun 5”. Although the expression of *GmFLC-like* is upregulated under SD conditions, its relatively weak power cannot inhibit flowering accelerated by photoperiod. 

**Figure 7:**
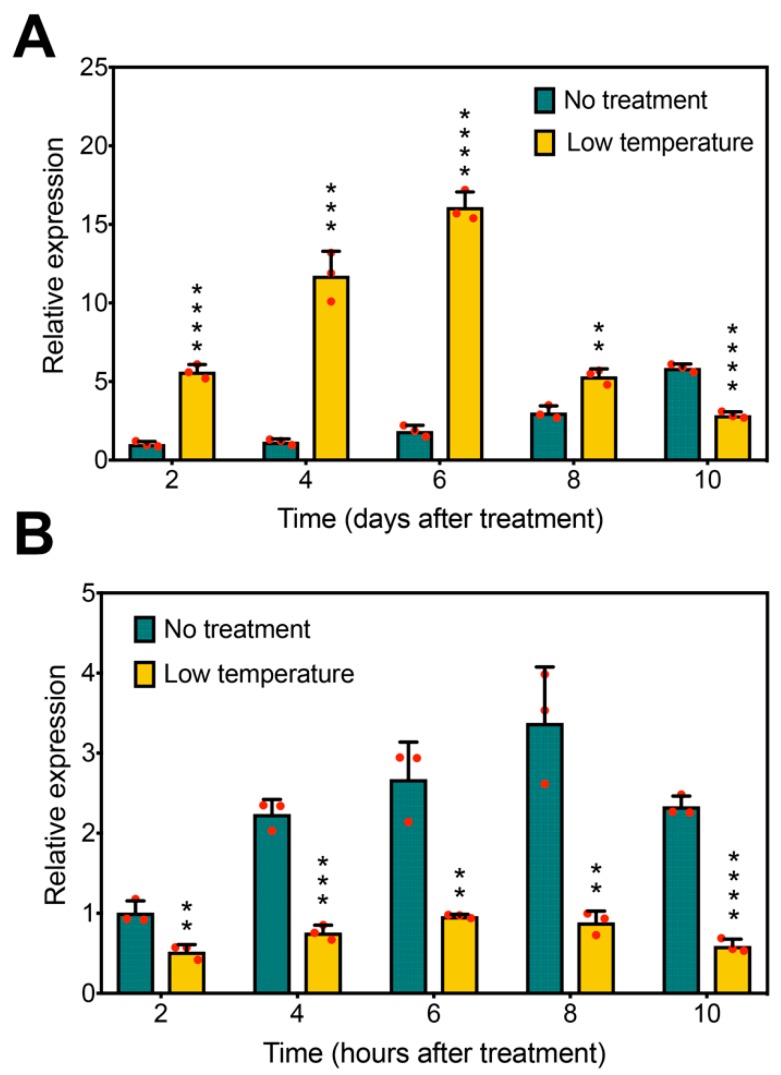
*GmFLC-like* response to photoperiod and low temperature. (**A**) Expression analysis of *GmFLC-like* at 2, 4, 6, 8, and 10 days after the beginning of low-temperature treatments and the control. (**B**) Expression analysis of *GmFLC-like* at 2, 4, 6, 8, and 10 h after the beginning of low-temperature treatments and the control. Error bars represent the means of three biological replicates. Significant differences according to the *t*-test are denoted as follows: ** *p* < 0.01, *** *p* < 0.001, **** *p* < 0.0001.

In Arabidopsis, *AtFLC* functions as a floral repressor and is a key gene responding to low temperature [29,51,52]. Our previous research showed that long-term low-temperature treatment resulted in delayed flowering in soybean [46]. To investigate whether *GmFLC-like* is responsive to low temperature, we tested *GmFLC-like* expression under low-temperature treatment by qRT-PCR. With respect to a long-term low-temperature treatment, *GmFLC-like* expression was significantly higher in treated plants than in controls after treatment for 2, 4, 6, and 8 days. Surprisingly, compared with the untreated plants, *GmFLC-like* expression was decreased in the treated ones after 10 days treatment (Figure 7A).

In contrast, short-term low-temperature treatment does not trigger the phenotype of delayed flowering. *GmFLC-like* expression also showed a remarkable difference when subjected to short-term low-temperature treatment. Compared with the controls, the plants displayed significantly lower *GmFLC-like* expression when subjected to a short-term low-temperature treatment for 2, 4, 6, 8, and 10 h (Figure 7B). Together, these results suggest that the expression pattern of *GmFLC-like* is clearly different between long-term and short-term low-temperature treatments. In other words, *GmFLC-like* may play a role in long-term low temperature-triggered delayed flowering in soybean based on its expression being strongly upregulated by long-term low-temperature treatment and downregulated by short-term low-temperature treatment.

### 2.5. Identification of GmFT2a as a Downstream Target of GmFLC-like

**Figure 8:**
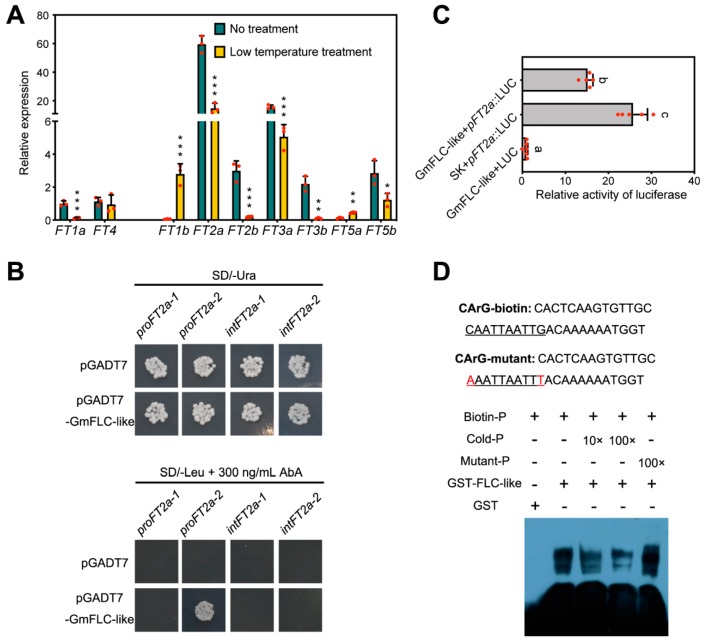
GmFLC-like protein binds to the promoter region of *FT2a*. (**A**) Expression analysis of *GmFTs* genes in soybean after the beginning of low-temperature treatments at 8 DAE. The soybean accession numbers are as follows: *GmFT1a* (*Glyma18g53680*), *GmFT4* (*Glyma08g47810*), *GmFT1b* (*Glyma18g53690*), *GmFT2a* (*Glyma16g26660*), *GmFT2b* (*Glyma16g26690*), *GmFT3a* (*Glyma16g04840*), *GmFT3b* (*Glyma19g28390*), *GmFT5a* (*Glyma16g04830*), and *GmFT5b* (*Glyma19g28400*). *Gmβ-tubulin* (*Glyma20g27280*) was used as an internal control. The mean values ± SD from three biological replicates are shown. Significant differences according to the *t*-test are denoted as follows: ** *p* < 0.05, ** *p* < 0.01, *** *p* < 0.001. (**B**) Interaction of GmFLC-like protein and *CmFT2a* promoter and the intron region, as revealed using a yeast one-hybrid system. The yeast transformations were plated onto SD/-Ura (upper panel) and SD/-Leu containing 300 ng/mL AbA (lower panel). pGADT7 with pAbAi-proFT2a-1 (from −1275 to −1156 bp), pAbAi-proFT2a-2 (from −671 to −552 bp), pAbAi-intFT2a-1 (from 410 to 551 bp), and pAbAi-intFT2a-2 (from 654 to 841 bp), were used as negative controls. The experiment was performed independently three times. (**C**) Relative reporter activity (LUC/REN) in *N. benthamiana* leaves. the relative luciferase activity (LUC/REN) in tobacco leaves were measured after 48 h of *Agrobacterium* infiltration. Experiments were repeated five times and mean value ± SD is plotted on the graph. The letters indicate significant differences according to Duncan’s multiple range test (*p* < 0.05). (**D**) Gel-shift analysis of GmFLC-like binding to the promoter region of *GmFT2a*. The sequence fragment from −663 to −628 of the *GmFT2a* promoters was used as a probe, and the core sequences are underlined. Purified protein (3 µg) was incubated with 25 picomoles biotin-labeled probe. For competition test, non-labeled probes at varying concentrations (from 10- to 100-fold excess), and mutated unlabeled CArG probe were added to the above experiment.

In Arabidopsis, *FLC* is responsible for regulating floral activator *FT* expression. *FLC* suppresses flowering mainly by repressing the expression of these floral activators [25,26,27]. According to a previous study [53] and the newest information from the Phytozome database, we discovered nine *FT* homologues, including *FT1a*, *FT1b*, *FT2a*, *FT2b*, *FT3a*, *FT3b*, *FT4*, *FT5a*, and *FT5b*. The flowering-inhibiting genes *FT1a* and *FT4* exhibited higher accumulation under LD, and represented opposite expression patterns of other FT genes [54]. To better understand whether *GmFLC-like* has a consistent function with *AtFLC*, we detected the expression profile of nine *FT* genes after the beginning of long-term low-temperature treatment for 8 days. QRT-PCR confirmed that, except for *FT1b* and *FT5a*, other *FT* homologues were downregulated after the beginning of long-term low-temperature treatment compared with the control (Figure 8A). In this study, we found that *GmFLC-like* expression was greatly elevated, and *GmFT1a*, *GmFT2a*, *GmFT2b*, and *GmFT2a* expression was significantly decreased after the beginning of the long-term low-temperature treatment. Previous research revealed that *GmFT2a* is responsible for inducing flowering under SD conditions, and the expression profiles of both *GmFLC-like* and *GmFT2a* were matched with the phenotype of delayed flowering (Figure 7A and Figure 8A). Next, we selected *GmFT2a* as a candidate gene for in-depth analysis to verify whether *GmFT2a* is a potential downstream target gene of *GmFLC-like*.

To better understand the regulatory mechanism between *GmFLC-like* and *GmFT2a*, a 1385-bp promoter region and a 792-bp first intron region of the *GmFT2a* sequence was identified. Sequence analysis using the New PLACE database (https://www.dna.affrc.go.jp/PLACE/?action=newplace) found that six CArG motifs (CWWWWWWWWG) exist in these sequences. Based on these findings, we designed the following experiments. First of all, we performed Y1H. The promoter sequence (−1275 to −1156 bp; −671 to −552 bp) and the first intron region (410 to 551 bp; 654 to 841 bp) of *GmFT2a* were amplified and inserted into the pAbAi vector, and the ORF sequence of *GmFLC-like* was inserted into the pGADT7 plasmid. The yeast one-hybrid (Y1H) assay confirmed that the GmFLC-like protein could target the promoter region (−671 to −552 bp) instead of the first intron region of *GmFT2a* by observing the transformants growth on SD/-Leu supplemented with 300 ng/mL AbA (Figure 8B). Additional evidence from a dual-luciferase reporter assay in *N. benthamiana* revealed that the activity of the promoters of *GmFT2a* could be inhibited by overexpression of GmFLC-like driven by the CaMV 35S promoter (Figure 8C). EMSA was also performed to verify the binding of GmFLC-like to the *GmFT2a* promoter. The 36 bp sequence fragment spanning positions −663 to −628 of the GmFT2a promoter was used as probe. The probe contained the predicted CArG motif (CAATTAATTG), which is the binding site for plant MADS domain protein. The fusion protein GST-GmFLC-like was purified from *Escherichia coli*, and then co-incubated with biotin-labeled and non-labeled probes. Finally, GmFLC-like was found to bind to the biotin-labeled proGmFT2a probe, furthermore, binding capacity was slowly weakened by increasing concentrations of non-labeled probe, while it was not affected by mutated unlabeled GCC probe (Figure 8D). These results indicated that *GmFT2a* was potentially one of the direct targets of GmFLC-like.

## 3. Discussion

For plants, temperature is a main environmental cue that has a strong influence on flowering time through different pathways, which mainly including vernalization and ambient temperature pathways [1,4,55]. In many species, *FLC*, encoding a MADS-box transcription factor, is the core gene in the vernalization way, which prevents flowering through inhibiting several floral activators, including *FT* and *SOC1* [13]. In *Brassica* plants, *FLC*s have been well studied over many years [56,57,58]. The researchers found that vernalization is closely correlated with *FLCs* epigenetic silencing, such as antisense RNA COOLAIR. The transcripts of *COOLAIR* are polyadenylated at multiple sites with proximal polyadenylation promoted by components of the autonomous promotion pathway. Use of the proximal poly(A) site results in quantitative downregulation of *FLC* expression in a process requiring FLD, an H3K4me2 demethylase [59]. Furthermore, the functions of *FLC* homologues show common features among different species [31,34]. However, no functional *FLC* homologues have been characterized in crops thus far [60]; one possible reason for this is that some crops have lost their *FLC* homologues. For example, *FLC* homologues may be absent from the rice genome [61]. For winter cereals, a gene called *VRN2*, which is downregulated in vernalization and functions in inhibiting *FT* expression [62], plays a similar role instead of genes orthologous to *FLC*. 

Soybean is considered a typical short-day plant that does not require vernalization. Interestingly, it still retains only one homologue of *FLC*, *Glyma05g28130*by comparative genomic analysis [63,64]. In our previous study, we found that *Glyma05g28130* were strongly upregulated in delayed-flowering *AtDREB1A*-overexpressing transgenic soybean [45]. Hence, we speculate that *Glyma05g28130* is involved in flowering time regulation. In the paper, we found *Glyma05g28130* belongs to a member of the MADS-box family transcription factor, containing two conserved domains, the MADS-box domain and the K domain (Figure 1A). Phylogenetic analysis revealed that *Glyma05g28130* falls within the FLC clade and is closely related to *AtFLC* in Arabidopsis, so named *GmFLC-like* (Figure 1B). In addition, the MADS-box domain between the GmFLC-like and FLC proteins from other species shared high conservation (Figure 2). These cues imply that *GmFLC-like* may also be a floral repressor and responsible for directly targeting and repressing the expression of floral activators *FT*, showing similar functions with Arabidopsis [25,26,27]. Then, heterogeneous overexpression of this gene in Arabidopsis indeed resulted in a marked late-flowering phenotype compared with the WT (Figure 5B,C). In addition, the expression of *SOC1*, *AP1*, and *FT* was significantly decreased in transgenic Arabidopsis (L48 and L46) (Figure 5D). Previous studies also confirmed that *AtFLC* homologues, such as *BvFL1* and *CiFL1*, perform the same functions in different species [23,29,30,34,65,66]. In summary, these findings suggest that some biological functions of *AtFLC* homologues are conserved among different species.

For vernalization-requiring plants, the transcripts of *FLC* are very high before vernalization but are subsequently inhibited by epigenetic modification under cold conditions and further accelerate flowering. While for soybean, a non-vernalized plant, shows the contrary phenomena. We found that the popular variety “Huachun 5”, which is suitable for growing in south china, shows sensitivity to low temperature. Under the long-term low-temperature treatment, *GmFLC-like* expression was induced and attributed to the delayed-flowering phenotype (Figure 7A), As we know, the soybean originated from north china, where the temperature is low. Therefore, we speculate that the soybean genome retains the low-temperature sensitive genes in order to cope with disadvantageous environments. Afterwards, to enlarge the planting range of soybean, the breeders selected out the varieties which are available to grow in early spring of south china. Meanwhile, the low-temperature-responsive gene *FLC* may be retained under the selection process for fitting the relative low temperatures.

In Arabidopsis, FLC delays the flowering time by repressing the expression of the floral integrator gene *FT* [12,13,25,67]. In this study, we found that *GmFLC-like* expression was greatly increased, meanwhile the *GmFT2a* expression was significantly decreased under the long-term low-temperature treatment (Figure 7A and Figure 8A), the results imply that the FLC-FT model may exist in soybean as well. Afterwards, we confirm that the GmFLC-like protein binds to the promoter region of *GmFT2a* promoter in vivo and vitro (Figure 8B−D). The FLC-FT2a model is confirmed, revealing that the molecular mechanism of flowering time is conversed among different species once again. Besides the existing variability in the FT2a-FLC model, these results imply that *GmFLC-like* may play an important role in delaying flowering time and providing protective mechanisms against sporadic and extremely low temperatures. 

## 4. Materials and Methods

### 4.1. Plant Materials and Growth Conditions

The soybean cultivar “Huachun 5”, which was bred by the Guangdong Sub-center of the National Center for Soybean Improvement, was used as the experimental material. Mature soybean seeds were surface sterilized and germinated in vermiculite. Uniform soybean seedlings were selected and grown in plastic pots containing turf soil and vermiculite at a ratio of 3:1 (*v/v*) in a growth chamber at 27 ± 2 °C, 40% relative humidity, and 100 μmol m^−2^ s^−1^ illumination with fluorescent lamps. Different regimes of day length as following: SD conditions (8 h of light/16 h of dark) and LD conditions (16 h of light/8 h of dark). The Arabidopsis wild-type (WT) and transgenic plants were of the Columbia (Col-0) ecotype. Arabidopsis seeds were surface sterilized and sown on half-strength MS medium for 2 days at 4 °C to relieve dormancy. Subsequently, the plates were transferred to a growth chamber at 22 ± 1 °C under LD conditions (16 h of light/8 h of dark) for 7 days, and then seedlings were transferred to pots containing turf soil and vermiculite (*v/v* 3:1).

### 4.2. Total RNA Isolation and qRT-PCR Analysis

The total RNA of the second trifoliate soybean leaf was isolated using TRIzol reagent (Invitrogen, Carlsbad, CA, USA). Then, 1 μg of total RNA was reverse-transcribed using HiScript II Q RT SuperMix for qPCR (R233-01, Vazyme Biotech Co., Nanjing, China). QRT-PCR analysis was carried out on a StepOne Real-Time PCR System (Applied Biosystems, Foster, CA, USA) using a Kapa SYBR Fast Universal qPCR Kit (Kapa Biosystems, Boston, MA, USA). Soybean *β-tubulin* (Glyma20g27280) and Arabidopsis *TUB2* (AT5G62690) were used as internal controls. The experiments were performed in three biological replicates, and the data were evaluated by the 2^−ΔΔCt^ methods [68]. All primers used in this study are listed in Table S1.

### 4.3. Plasmid Construction

The open reading frame (ORF) of *GmFLC-like* was amplified using specific primers K-FLC-F and K-FLC-R and inserted into the pCAMBIA1301 plasmid by the *Bam*HI and *Kpn*I restriction sites to form the expression vector *35S::GmFLC-like-GFP*. Genomic DNA was isolated from leaf samples of soybean using the modified cetyltrimethyl ammonium bromide (CTAB) method [69]. The promoter sequence of *GmFLC-like* was amplified using specific primers designed as Pro-GmFLC-F and Pro-GmFLC-R, and genomic DNA of soybean was used as the template. The amplified PCR product was inserted into a pZeroBack/blunt vector (Tiangen Biotech Co., Beijing, China) for sequencing. All primers used are listed in Supplementary Table S1.

### 4.4. Sequence Analysis

The conserved domains of GmFLC-like were predicted by using InterProScan [70]. The phylogenetic tree was generated based on alignment results using the neighbor-joining algorithm with 1000 bootstrap replicates in MEGA 5.0 software [71]. Multiple amino acid sequences were aligned using ClustalW with default parameters, and the comparison result was displayed by the software BioEdit [71]. Protein sequence logos were created using WebLogo 3.3 [72]. Homologous protein sequences of GmFLC-like were searched in the Phytozome databases [47]. The *cis*-acting elements in the *GmFLC*-*like* promoter (1000 bp upstream of the start codon) were analyzed using the PlantCARE program [73].

### 4.5. Subcellular Localization of GmFLC-like Protein

The ORF sequence of GmFLC-like without the stop codon was fused to the N-terminal region of the GFP protein, and the resulting fragment was inserted into the pCAMBIA1301 plasmid to form the expression vector *35S::GmFLC-like-GFP*. The fused vector was transformed into epidermal cells of *Nicotiana benthamiana* leaves by *Agrobacterium* infection and expressed for 3 days. The fluorescence signals were monitored by confocal microscope (Olympus FluoView FV1000). The GFP and mCherry protein were imaged using 488 and 543 nm excitation, respectively. The plasmids *35S::GFP* and *35S::NF-YA4-mCherry* were used as the negative control and nuclear marker, respectively [74].

### 4.6. Ectopic Expression of GmFLC-like in Arabidopsis

The expression vector *35S::GmFLC-like-GFP* was transformed into the *Agrobacterium tumefaciens* GV3101 strain by electroporation. Arabidopsis (Col-0) was used for transgenic material and performed using the floral dip method [75]. Transgenic Arabidopsis seeds were grown on half-strength MS medium supplemented with 25 mg/L hygromycin, and three homozygous T_2_ transgenic lines with different *GmFLC-like* expression levels were chosen for further studies, including phenotype investigation and expression analyses of potential downstream genes.

### 4.7. Photoperiod Treatment

Soybean seeds were grown under SD conditions for 10 days, and then, part of uniform seedlings was transferred to LD conditions. Fully expanded trifoliate leaves were sampled at 12, 15, 18, 21, 24, 27, and 30 DAE from plants growing under SD and LD conditions, respectively. All samples were immediately frozen in liquid nitrogen and stored at −80 °C for further study.

### 4.8. Low-Temperature Treatment

Uniform soybean seedlings were grown in a growth chamber at 28 °C/26 °C (day/night) under SD conditions until the fourth trifoliate stage. Then, the seedlings were divided into two groups for the following low-temperature treatments: the long-term treatment group and the short-term treatment group. For the long-term treatment group, plants were continuously treated at 15 °C/13 °C (day/night) under SD conditions for 10 days and leaves were sampled every 2 days from three individual plants. For the short-term treatment group, plants were continuously treated at 15 °C/13 °C (day/night) for 10 h in the darkness, and leaves were sampled every 2 h from three individual plants. The control seedlings were grown at 28 °C/26 °C under the same conditions corresponding to the long- and short-term treatment groups. Leaves were sampled from three individual control plants every 2 days at the same collection time used for the low temperature-treated plants.

### 4.9. Yeast One-Hybrid Assay

A yeast one-hybrid assay (Y1H) assay was performed as previously described [76,77]. The promoter and the first intron sequence of *GmFT2a* were amplified and inserted into the pAbAi vector to form the bait plasmid, and the obtained bait plasmids were recognized as pAbAi-proFT2a-1, pAbAi-proFT2a-2, pAbAi-intFT2a-1, pAbAi-intFT2a-2, respectively. After linearization by *BstB*I, the bait plasmids were transformed into yeast strains Y1H Gold according to the manufacturer’s instruction of the Matchmaker^TM^ Gold Yeast One-Hybrid Library Screening System Kit (Clontech, Mountain View, CA, USA). The *GmFLC-like* ORF sequence was inserted into the pGADT7 vector to form the prey plasmid. The prey plasmid was co-transformed into yeast strain Y1Hgold. Subsequently, the yeast transformants were plated onto synthetic dropout (SD) medium lacking uracil (Ura) or leucine (Leu) but supplemented with 300 ng/mL Aureobasidin A (AbA). After 3 days of incubation in a 28 °C incubator, the interactors were defined on the basis of transformants growth on SD/-Leu medium with 300 ng/mL AbA.

### 4.10. Dual-Luciferase Reporter Assay

For dual-luciferase assay, we introduced two vectors: pGreenII 0800-LUC and pGreenII 0029 62-SK. The *GmFT2a* promoter was amplified and inserted into the pGreenII 0800-LUC vector to drive the LUC reporter gene expression, and the GmFLC-like ORF sequence was inserted into the pGreenII 0029 62-SK vector. The pGreenII 0800-LUC vector carries a renilla luciferase (REN) gene driven by the 35S promoter to serve as an internal control. The two plasmids were co-expressed into tobacco leaves by Agrobacterium infiltration as previously described [72]. At 48 h after transformation, LUC and REN luciferase activities were measured using a Dual-Luciferase^®^ Reporter Assay System (Promega, Madison, WI, USA) and a Glomax^®^-20/20 signal Tube Luminometer (Promega, Madison, WI, USA). The relative activity of luciferase was calculated based on the ratio of the luciferase activity of the sample to that of the *Renilla* luciferase control.

### 4.11. Electrophoretic Mobility Shift Assay

Briefly, the *E. coli* BL21 cells carrying the GST-GmFLC-like fusion protein was incubated in 100 mL LB medium supplemented with isopropyl IPTG to achieve the rated concentration (0.2 mM), and the cell cultures were incubated at 30 °C for 6 h. The fusion protein was extracted from *E. coli* cells and purified according to the manufacturer’s instructions of glutathione beads (Glutathione-Sepharose 4B, GE Healthcare, Chicago, IL, USA). EMSA was performed according to the description of the LightShift^®^ Chemiluminescent EMSA Kits (Pierce, Milan, Italy). The biotin-labeled DNA labeled fragments (5′-CACTCAAGTGTTGCCAATTAATTGACAAAAAATGGT-3′) were synthesized, annealed, and used as probes, with unlabeled DNA of the same sequence and mutant sequence (5′-CACTCAAGTGTTGCAAATTAATTTACAAAAAATGGT-3′) used as the competitors.

### 4.12. Data Analysis

For data analysis and graphs generation, GraphPad Prism 8.0 (GraphPad Software Inc., San Diego, CA, USA) was used. For comparison of two groups of data, the two-sided Student’s *t*-test was used, thereinto, asterisks *, **, ***, and **** indicate significant differences at *p* < 0.05, *p* < 0.01, *p* < 0.001, and *p* < 0.0001, respectively. For comparison of multiple groups of data, one-way ANOVA and Bonferroni’s post hoc test was used, and *p* < 0.05 was considered significant.

### 4.13. Data Availability

The GenBank accession numbers for soybean genes used in this study are as follows: GmFLC-like (MK913903); Promoter of GmFLC-like (KY203812).

## Figures and Tables

**Table 1:** *cis*-acting elements of the *GmFLC-like* promoter.

*cis*-Element	Sequence (5′-3′)	Position (From ATG)	Description
**Light regulation elements**			
AE-box	AGAAACTT	-1089(-)	Part of a module for light response
CATT-motif	GCATTC	-1467(-),-517(-)	Part of a light responsive element
G-box	TACGTG	-134(-)	*cis*-acting regulatory element involved in light responsiveness
TCT-motif	TCTTAC	-425(-)	Part of a light responsive element
AT1-motif	AATAATTTTTTATT	-915(+)	Part of a light responsive module
Box 4	ATTAAT,TA(C/A)TTA	-1259(+),-498(+),-169(+),-574(+),-285(+)	Part of a conserved DNA module involved in light responsiveness
Box I	TTTCAAA	-581(-),-506(-)	Light responsive element
G-Box	CACGT(T/A)	-790(+),-117(+)	*cis*-acting regulatory element involved in light responsiveness
as-2-box	GATAATGATT	-657(-)	Involved in shoot-specific expression and light responsiveness
rbcS-CMA7a	GGCTATAAGG	-104(+)	Part of a light responsive element
chs-CMA1a	TTACTTAA	-575(-)	Part of a light responsive element
**Hormone and development-related elements**			
Circadian	CAANNNNATC	-86(-)	*cis*-acting regulatory element involved in circadian control
Skn-1_motif	GTCAT	-148(+),-1396(+)	*cis*-acting regulatory element required for endosperm expression
ABRE	(C/T)ACGTG	-139(+),-112(-)	*cis*-acting element involved in the abscisic acid responsiveness
GARE-motif	TCTGTTG	-1071(-)	gibberellin-responsive element
TCA	GAGAAGAATA,CCATTTTTTT	-1162(-),-751(-)	*cis*-acting element involved in salicylic acid responsiveness
AuxRR-core	GGTCCAT	-748(-)	*cis*-acting regulatory element involved in auxin responsiveness
**Abiotic stress response elements**			
HSE	AAAAAATTTA	-423(+)	*cis*-acting element involved in heat shock responsiveness
MBS	(C/T)AACTG	-1081(-),-649(+)	MYB binding site involved in drought-inducibility
ARE	TGGTTT	-1131(+)	*cis*-acting regulatory element essential for the anaerobic induction
CE3	AACGCGTGTC	-1330(+)	*cis*-acting element involved in ABA and VP1 responsiveness

## Supplementary Materials

Supplementary materials can be found at https://www.mdpi.com/1422-0067/21/4/1322/s1. Table S1. Primers used in this study; Figure S1. Subcellular localization of GmFLC-like protein in Arabidopsis protoplast cells.; Figure S2. Comparative analysis of seed germination rate among four lines of Arabidopsis.

## Abbreviations

LD	long-day
SD	short-day
FLC	flowering locus C
Y1H	yeast one-hybrid
EMSA	electrophoretic mobility shift assay
FT	flowering locus T
FRI	frigida
VIN3	vernalization insensitive 3
COOLAIR	cold induced long antisense intragenic RNA
COLDAIR	cold assisted intronic noncolding RNA
SOC1	suppressor of overexpression of constants 1
VRN1	vernalization 1
VRN2	vernalization 2
AP1	apetala 1
MS	murashige and skoog
TUB2	tubulin beta chain 2
AbA	aureobasidin A
IPTG	β-d-1-thiogalactopyranoside
