# Supplementary material for: The Floral Repressor GmFLC-like Is Involved in Regulating Flowering Time Mediated by Low Temperature in Soybean
Source: Int J Mol Sci. 2020 Feb 15;21(4):1322. doi: 10.3390/ijms21041322 (PMC7072909; doi:10.3390/ijms21041322)
Supplement: Supplementary file 1 [file ijms-21-01322-s001.pdf]

Supplementary Information

The floral repressor *GmFLC-like* is involved in regulating flowering time mediated by low temperature in soybean

This PDF file includes:

Supplementary Fig. 1-2

Supplementary Table 1

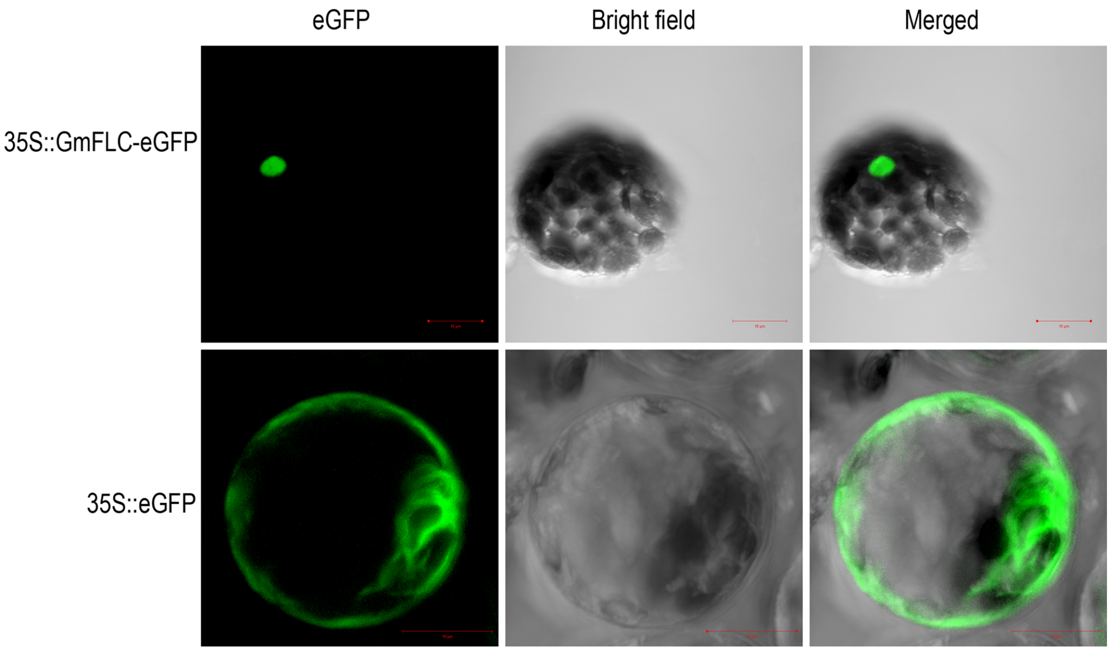

Fig. S1: Subcellular localization of GmFLC-like protein in Arabidopsis protoplast cells.

Constructs 35S::*GmFLC-like-eGFP* were transformed into Arabidopsis protoplast cells. The Empty plasmid *35S::eGFP* was used as a control. The cells were observed under a confocal laser microscope. Scale bars, 10 μm.

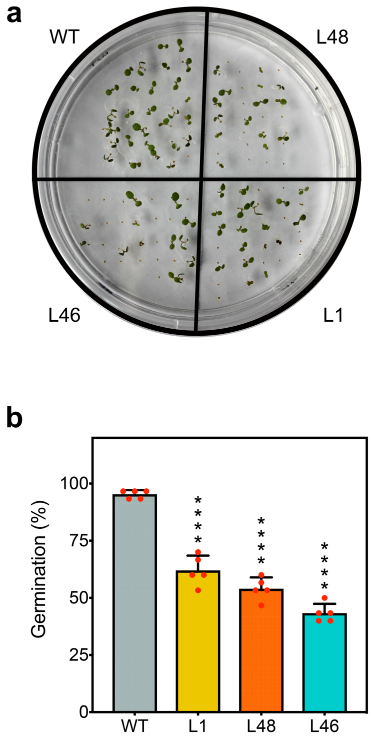

Fig. S2: Comparative analysis of seed germination rate among four lines of Arabidopsis. a Germinating status of WT (Col-0) and three *GmFLC-like* transgenic lines L1, L46, and L48 seeds on 1/2 MS medium at 3 days after transfer to light. **b** Seed germination rate of WT and three transgenic lines on 1/2 MS medium. Germination percentage was counted for approximately 30 seeds for each line. Experiments were repeated five times and mean value ± SD is plotted on the graph. Significant differences according to the *t*-test are denoted as follows: * $p < 0.05$, ** $p < 0.01$, *** $p < 0.001$, **** $p < 0.0001$.

Supplementary Table 1. Primers used in this study

Primers	Sequence
**For gene isolation**	
K-FLC -F	ATGGGGAAGAAGAAGCTGGAGAT
K-FLC-R	CAACCTCTACCACTAGGCCAATCAT
**For vector construction of overexpression in Arabidopsis**	
1301-FLC -F	CGCGGATCC(BamHI)ATGGGGAAGAAGAAGCTGG
1301-FLC -R	CGGGGTACC(KpnI)TTATTTATTTATACTGAGTTCAAGAATT
**For vector construction of subcellular localization**	
FLC-GFP -F	CGGGGTACC(KpnI)ATGGGGAAGAAGAAGCTGG
FLC-GFP -R	CGGGATCC(BamHI)TTTATTTATACTGAGTTCAAGAATTGAG
**For gene promoter isolation**	
Pro-GmFLC -F	TTGCTTCGGTTACTGTTTCTTCC
Pro-GmFLC -R	TGTTCTCGATTCGCTTTATCTCC
proFT2a-1-F	ATTGGTACCCCGGGTGGGGAAGGGCTACT
proFT2a-1-R	ATTCTCGAGAACATTTCCTTCCCTTCTC
proFT2a-2-F	ATTGGTACCTCCTTTTTTCACTCAAGTG
proFT2a-2-R	ATTCTCGAGTTACTTATTTAATGGAAACTA
intFT2a-1-F	ATTGGTACCTATGATTTTAGTTTCATT
intFT2a-1-R	ATTCTCGAGTAATGGATGCTATATCAT
intFT2a-2-F	ATTGGTACCTTATTTATCTATCTCTTTT
intFT2a-2-R	ATTCTCGAGTGACTTTAAGTCCTATAAAA
**For transgenic plants confirmation**	
Hpt -F	ACTTCTACACAGCCATCGGTCC
Hpt -R	AGCGAGAGCCTGACCTATTGC
**For qRT-PCR analysis**	
Gm-FLC -F	TGACGCATAATCTGCTCCCTG
Gm-FLC -R	GCTAAACCATGGCATAGTTCCCT
Gm-β-Tublin -F	CCTCGTTCGAATTCGCTTTTTG
Gm-β-Tublin -R	CAACTGTCTTGTCGCTTGGCAT
GmFT1a -F	CCTTTTACACCCTGGTTATGG
GmFT1a -R	CCTGGAGGTTGCAGAGTTAGT
GmFT1b -F	GACTTCAGGACCTTTTACACCC
GmFT1b -R	GCTCACAACCTCTTCACCGA
GmFT2a -F	ATCCCGATGCACCTAGCCCA
GmFT2a -R	ACACCAAACGATGAATCCCCA
GmFT2b -F	GACATTCCAGCAACAACGG
GmFT2b -R	ATAGCCTTCTTCCACCACAAC
GmFT3a -F	GGATTCATCGTTTCGTGTTTG
GmFT3a -R	CACCAGAGCCAGTTTCCCT
GmFT3b -F	CTATGAAAGCCCACGACCC
GmFT3b -R	TTGAAGAAGACAGCAGCAACC
GmFT4 -F	GTGAGTTCAAACCTTCCCAAAT
GmFT4 -R	CAATCCGATGAATCCCAGAA
GmFT5a -F	ACAGATTATGGTAGCAACGGAA
GmFT5a -R	CAAGGATAGCCAGAAAAGAAAG

GmFT5b -F	CTCAATCCTTTTACAATCTCCG
GmFT5b -R	CCTTAGGTCTTCACCACCAACA
At-FT-F	CCCTGCTACAACTGGAACAAC
At-FT-R	AAGAACAAGGTAACCCAATGAAC
At-SOC1 -F	AAACGAGAAGCTCTCTGAAAAG
At-SOC1 -R	AAGAACAAGGTAACCCAATGAAC
At-AP1-F	GCAAGCAATGAGCCCTAAAG
At-AP1-R	ACTGCTCCTGTTGAGCCCTA
At-TUB2 -F	ATCGATTCCGTTCTCGATGT
At-TUB2 -R	ATCCAGTTCCTCCTCCCAAC
